# Familial occurrence of concomitant imperforate hymen and transverse vaginal septum: a case report

**DOI:** 10.11604/pamj.2022.42.207.33418

**Published:** 2022-07-15

**Authors:** Zineb Aboueddahab, Khalid Guelzim, Jaouad Kouach

**Affiliations:** 1 Department of Gynecology and Obstetrics, Military Instruction Hospital Mohammed V, Rabat, Morocco,; 2 Faculty of Medicine and Pharmacy of Rabat, Mohammed V university, Rabat, Morocco

**Keywords:** Female reproductive tract anomalies, imperforate hymen, hematometrocolpos, familial, case report

## Abstract

The imperforate hymen and transverse vaginal septum are female reproductive tract abnormalities. Their concurrent occurrence is rare. So far, no manifestation of both anomalies within the same family has been reported in the literature. We report the first familial case of two sisters having both mentioned abnormalities. Our patient was the youngest sister of a 14-year-old, who presented with cyclic lower abdominal pain. Clinical and imaging examinations showed an imperforate hymen with hematometrocolpos. In addition, the surgery confirmed a low transverse vaginal septum, which was resected using a virginity-preserving procedure. In conclusion, the presence of both mentioned abnormalities between siblings reflects a potential genetic etiology, also in order to avoid late diagnostics of such anomalies, the imperforate hymen must be screened in the birth room.

## Introduction

Female reproductive tract anomalies are often discovered by the evaluation of menstrual complications or fertility disorders. These anomalies are common. They present an incidence rate of 2% to 3% [1]. The incidence of the imperforate hymen is estimated between 0.014% and 0.1% [2]. While the transverse vaginal septum is rarer, with an incidence rate of 0.001% [2]. Being diagnosed with both mentioned anomalies can generate an obstruction of the female genital tract, leading to primary amenorrhea together with a hematometrocolpos [2]. The occurrence of both abnormalities is unique [1]. In the literature, only five worldwide cases report on concurrent imperforate hymen and transverse vaginal septum [2]. None of these cases describe a manifestation of both obstructive lesions within the same family. In this paper, we report the first familial case of concurrent imperforate hymen and transverse vaginal septum in the literature.

## Patient and observation

**Patient information:** a 14-year-old girl with no personal medical or surgical history presented with primary amenorrhea and abdominal pain. For family history, the oldest sister was diagnosed with an imperforate hymen with transverse vaginal septum four years ago.

**Figure 1:**
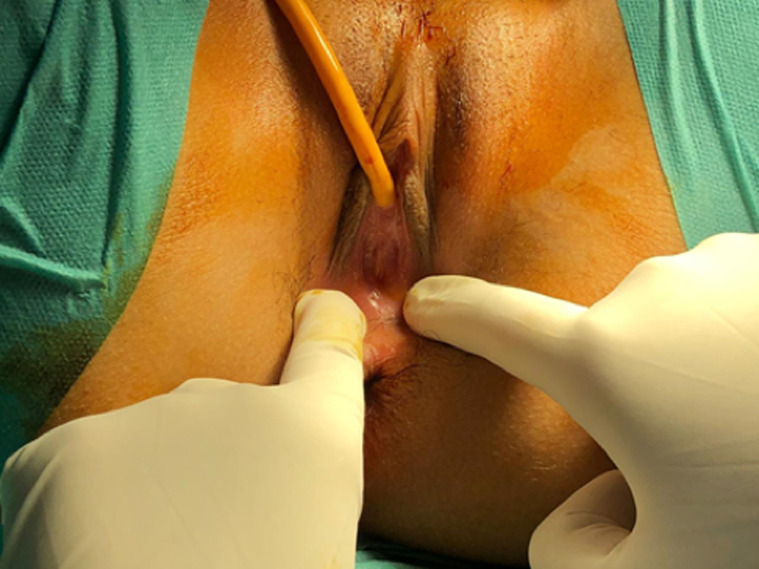
image of vulva showing imperforate hymen

**Clinical findings:** during the examination, it was observed that the patient had tanner stage IV breast and pubic hair, as well as, normal-appearing labia majora and minora. Also, while examining the external genitalia, an absence of a hymenal opening without a bulge or bluish discoloration of hymen was revealed, as it can be seen in (Figure 1). Moreover, the rectal examination showed a tender soft mass palpated in the upper and the medium part of the vagina.

**Timeline of the current episode:** the symptomatology, which had occurred on approximately five occasions during the past six months.

**Figure 2:**
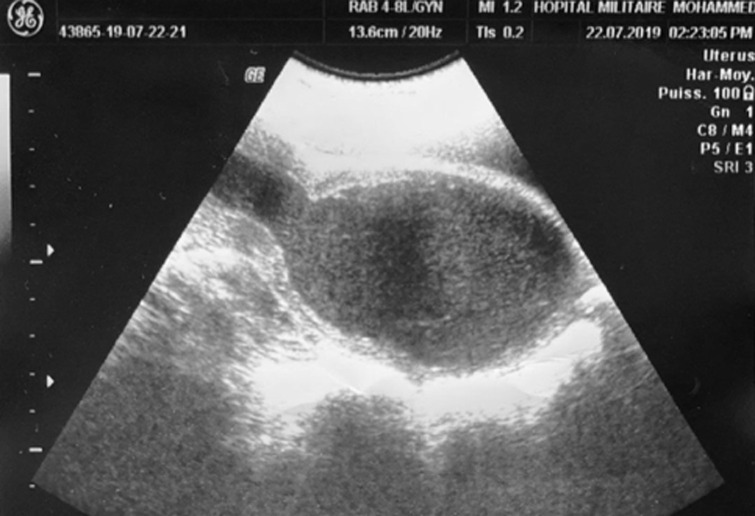
sagittal midline ultrasound scan of pelvis showing a large hematocolpos with hematometra

**Figure 3:**
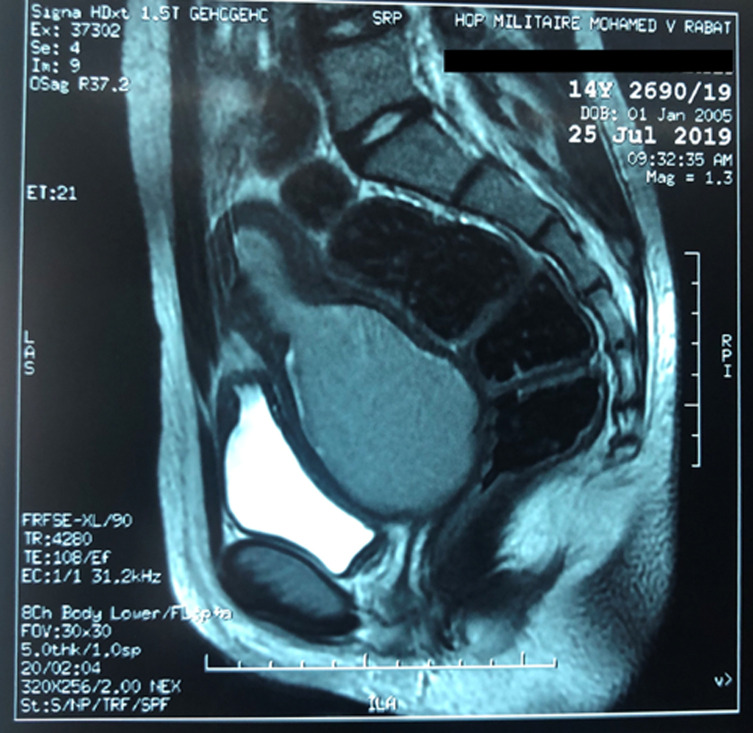
magnetic resonance imaging (MRI) showing hematometra and hematocolpos with obstruction at the lower third of the vagina

**Diagnostic assessment:** an ultrasound scan showed the presence of hematometra and hematocolpos with bilateral hematosaplinx (Figure 2). Further evaluation by magnetic resonance imaging (MRI) confirmed the ultrasound results, in addition to the association of a narrowing of the lower third of the vaginal canal (Figure 3), without differentiating between vaginal atresia and transverse vaginal septum. The checks did not reveal any further associated genitourinary anomalies.

**Diagnosis:** according to this, the diagnosis of imperforate hymen with transverse vaginal septum was retained.

**Therapeutic interventions:** in this case, a surgery was indicated. Under general anesthesia, an X-shaped hymenal incision was performed, and part of the tissue (all around) was kept for virginity preservation. After the incision, no drainage of blood or fluid was noticed. This led to performing a vaginoscopy that confirmed a complete transverse vaginal septum at the lower third of the vagina. This vaginal septum was excised by a bipolar electrode introduced through the operative channel of the hysteroscope, and about 800 ml of brown menstrual efflux was drained. Realizing, the hysteroscopy confirmed a normal cervix, an ostia bilaterally, and no intrauterine defects. The final stage of the surgery consisted of suturing the hymen´s edges and keeping a Foley catheter in the vagina above the septum level inflated with a saline, that was kept for 15 days.

**Follow-up and outcome of interventions:** the patient was followed for six months after the surgery. All along this period of time, we observed a complete resolution of symptoms as well as the appearance of regular cycles. Furthermore, the examination did not show stenosis of the hymen. Checking the medical records of the older sister showed that both sisters were subject to the same surgery and follow-up. In order to compare closely both cases, we summoned the older sister. Her check-up revealed that her hymen was still permeable.

**Patient perspective:** the patient was satisfied with the diagnostic and the proposed care.

**Informed consent:** patient gave his consent for his images and other clinical information to be reported in the journal.

## Discussion

By referring to female embryology, the lower part of the vagina and the hymen have the same embryological derivations. They are both derived from the urogenital sinus [3]. The failure of canalization results in the transverse vaginal septum and imperforate hymen. The exact amount of time needed for the hymen canalization remains unknown, and the stimulus of the hymeneal breakdown remains unclear [4]. In addition, the hymenal remodeling extends all along the third year after birth, resulting in the continuous growth of the hymenal opening [4]. The etiology of the reproductive tract anomalies remains anonymous [5]. However, the presence of a concomitant imperforate hymen and transverse vaginal septum between siblings, as referred to in this paper, suggests the existence of a genetic etiology. Up to now, only the TBX3 gene has been described in an imperforate hymen of a patient with an ulnar-mammary syndrome without the manifestation of a transverse vaginal septum [5]. By referring to the cases presented above, no one of them showed any additional physical abnormality. Inherited cases of imperforate hymen have been described in the literature. Both the recessive and the dominant modes of transmission have been suggested [5]. But nothing has been found related to the concurrence of both obstructive lesions presented in this paper between siblings. In our cases, these vaginal anomalies affected siblings, suggesting an autosomal recessive inheritance. The diagnosis of such abnormalities is usually delayed until after puberty like our two cases when female patients experience some complications such as; i) primary amenorrhea; ii) cyclic lower abdominal pain, and iii) mass. The imperforated hymen can be easily detected by examining the external genitalia, but it must be differentiated from a low transverse vaginal septum following the Valsalva manoeuver. The MRI is considered to be the gold standard for diagnosis [1]. Sonovaginography intraoperative can also be used for the diagnosis of transverse vaginal septum [6]. The treatment of these two obstructive lesions is surgical at one sitting. Several surgical techniques have been described in the literature. However, in most cultures, as for our patients, women desire to preserve their virginity until marriage. That is why we used a method that preserves the hymen. It combines two techniques described by two different authors [7,8]. First, is the preservation of the hymenal tissue during incision and suturing, in order to provide suitable ground for defloration during coitus. Second, using the vaginoscopic approach for the treatment of the transverse vaginal septum, to preserve the integrity of the hymen. The catheter balloon reduces the risk of stenosis, which is a potentially disabling complication.

## Conclusion

To sum up, our case demonstrates that the occurrence of both imperforate hymen and transverse vaginal septum can happen between siblings. This suggests the existence of a genetic etiology and most likely a recessive transmission. Routine postnatal screening of girls with a family history of these two abnormalities should be employed as a preventive measure against late diagnosis. Unless a large number of familial cases is studied, it remains difficult to prove surely the mode of transmission of these two abnormalities. Accordingly, further experience with a larger number of patients is necessary for analyzing genes and determining which one might be responsible for the manifestation of this phenomenon.
